# Induction of Systemic Resistance to *Tobacco mosaic virus* in Tomato through Foliar Application of *Bacillus amyloliquefaciens* Strain TBorg1 Culture Filtrate

**DOI:** 10.3390/v14081830

**Published:** 2022-08-20

**Authors:** Ahmed Abdelkhalek, Dalia G. Aseel, Lóránt Király, András Künstler, Hassan Moawad, Abdulaziz A. Al-Askar

**Affiliations:** 1 Plant Protection and Biomolecular Diagnosis Department, ALCRI, City of Scientific Research and Technological Applications, New Borg El Arab City 21934, Egypt; 2 Centre for Agricultural Research, Plant Protection Institute, ELKH, P.O. Box 102, H-1022 Budapest, Hungary; 3 Agriculture Microbiology Department, National Research Centre, Cairo 12622, Egypt; 4 Department of Botany and Microbiology, College of Science, King Saud University, P.O. Box 2455, Riyadh 11451, Saudi Arabia

**Keywords:** *Bacillus amyloliquefaciens*, TMV, oxidative stress, defense-related enzymes, gene expression, GC-MS

## Abstract

The application of microbe-derived products as natural biocontrol agents to boost systemic disease resistance to virus infections in plants is a prospective strategy to make agriculture more sustainable and environmentally friendly. In the current study, the rhizobacterium *Bacillus amyloliquefaciens* strain TBorg1 was identified based on *16S rRNA*, *rpoB*, and *gyrA* gene sequences, and evaluated for its efficiency in conferring protection of tomato from infection by *Tobacco mosaic virus* (TMV). Under greenhouse circumstances, foliar sprays of TBorg1 culture filtrate (TBorg1-CF) promoted tomato growth, lowered disease severity, and significantly decreased TMV accumulation in systemically infected leaves of treated plants relative to untreated controls. TMV accumulation was reduced by 90% following the dual treatment, applied 24 h before and after TMV infection. Significant increases in levels of total soluble carbohydrates, proteins, and ascorbic acid were also found. In addition, a significant rise in activities of enzymes capable of scavenging reactive oxygen species (PPO and POX), as well as decreased levels of non-enzymatic oxidative stress markers (H_2_O_2_ and MDA) were observed, compared to untreated plants. Enhanced systemic resistance to TMV was indicated by significantly increased transcript accumulation of polyphenolic pathway (*C4H*, *HCT*, and *CHI*) and pathogenesis-related (*PR-1* and *PR-5*) genes. Out of the 15 compounds identified in the GC-MS analysis, 1,2-benzenedicarboxylic acid mono(2-ethylhexyl) ester and phenol, 2,4-bis(1,1-dimethylethyl), as well as L-proline, N-valeryl-, and heptadecyl ester were present in the highest concentrations in the ethyl acetate extract of TBorg1-CF. In addition, significant amounts of n-hexadecanoic acid, pyrrolo [1,2-a] pyrazine-1,4-dione hexahydro-3-(2-methylpropyl)-, nonane, 5-butyl-, and eicosane were also detected. These compounds may act as inducers of systemic resistance to viral infection. Our findings indicate that the newly isolated *B. amyloliquefaciens* strain TBorg1 could be a potentially useful rhizobacterium for promoting plant growth and a possible source of biocontrol agents for combating plant virus infections.

## 1. Introduction

Climate change, coupled with global population growth and urbanization, contributes to the rising demand for increasing crop yields and enhanced food quality. Numerous initiatives have already been implemented to increase the rate of agricultural production. On the other hand, plant diseases cause considerable crop losses and stall advances in crop management [1,2]. Plant viral infections pose a significant danger to plant biosecurity, resulting in massive crop losses on a global scale [3,4]. *Tobacco mosaic virus* (TMV) is one of the most contagious plant diseases, owing to its broad host range (about 66 families with over 900 plant species) and severe infection consequences [5,6,7]. Mechanical transmission of TMV occurs when diseased plants, contaminated agriculture instruments, and/or contaminated seeds come into close physical contact [8]. In addition, TMV virions are exceptionally stable, ensuring persistence for years in infected materials such as fallen leaves or soil, with an intact potential for re-infection [9]. In tomatoes, various morphological abnormalities are caused by TMV infection, including systemic leaf mosaicism and necrosis, and leaf chlorosis [10]. Moreover, if TMV infection becomes more severe, it can cause systemic changes in the blooming organs, delaying fruit ripening, reducing crop output, and ultimately causing crop loss [11].

It is difficult to control TMV infection, depending primarily on adopting resistant plant cultivars and preventing vector spread by the intense application of insecticides, which can negatively affect the environment and public health [12]. The interest in biocontrol agents as environmentally benign alternatives for the toxic chemicals now used in plant pest management strategies is increasing to ensure long-term sustainability in agriculture and the environment [13,14,15]. Natural rhizosphere microbiota called plant growth-promoting rhizobacteria (PGPR) boost plant development and resistance to several diseases. Multiple studies have discovered that PGPR can promote plant growth while increasing resistance to, e.g., viral infection [16,17]. PGPRs promote plant growth by boosting nutrient absorption and producing biomolecules that are important in conferring stress tolerance. Depending on the circumstances, these activities may indirectly suppress the plant’s susceptibility to infections or combat pathogens directly by synthesis of antibiotics or competition for critical nutrients [18,19]. Strains of *Bacillus* spp., for example, are frequently used as PGPRs, their efficiency attributable to their diverse mechanisms for controlling infections and stimulating plant growth, which are mediated by, e.g., a large number of secondary metabolites [20,21]. Under field conditions, however, the survival and adaptation of PGPR inocula to natural microbiota are strain-specific and significantly influenced by procedures of application, which are frequently associated with ambiguous results in the laboratory [20]. Furthermore, it is unknown whether PGPR inocula impact the native microbial population at the application site, potentially increasing antagonistic resistance [22,23,24]. As a result of these drawbacks, microbial culture filtrates have been developed as an environmentally friendly and dependable alternative to PGPRs [20].

Generally, two basic mechanisms may cause induced resistance in plants. The first is systemic acquired resistance (SAR), which is induced by pathogens or elicitor molecules contacting aerial plant parts (leaves) and is controlled primarily by salicylic acid [7,25]. The second is induced systemic resistance (ISR), which is induced by PGPR (and other beneficial microorganisms) when they interact with plant roots, and is regulated by jasmonic acid and ethylene [25,26]. Both pathways contribute to plant viral resistance. The activation of both SAR and ISR results in numerous, partially overlapping cellular responses, including the activation of defense genes and antioxidant enzymes [25,27,28,29]. For example, the SAR pathway is characterized by the induction of defense genes, including those encoding for the pathogenesis-related proteins, PR-1, PR-2, and PR-5, while ISR is instead marked by elevated levels of PR-3, PR-4, PR-6, and PR-12.

The present study aimed to determine the efficacy of *Bacillus amyloliquefaciens* strain TBorg1-culture filtrate (TBorg1-CF) to enhance tomato growth and confer protection against TMV infection in tomato plants either directly or by inducing systemic resistance following foliar spraying. We assumed that the application of TBorg1-CF (instead of live bacteria) to tomato leaves (rather than roots) may imply, primarily, the induction of an SAR, rather than an ISR, against TMV. To elucidate the mechanisms of resistance induction, we determined the activities of enzymes involved in ROS scavenging (PPO, SOD, and POX), levels of non-enzymatic oxidative stress indicators (H_2_O_2_ and MDA), as well as DPPH (an indicator of free radical scavenging activity), ascorbic acid, and total carbohydrate and protein contents. In addition, the transcript levels of various defense-related genes, including *PR-1*, *PR-2*, *PR-5*, *C4H*, *HCT*, and *CHI*, were estimated in tomato tissues, along with TMV accumulation. Finally, the bioactive ingredients of TBorg1-CF were screened and identified using a gas chromatography-mass spectrometry (GC-MS) approach.

## 2. Materials and Methods

### 2.1. Plant Material and Viral Source

A virus-free line of the Carmen cultivar of tomato (*Solanum lycopersicum* L.), sensitive to TMV infection, was obtained from the Egyptian Agriculture Research Center. A purified isolate of TMV strain KH1 (accession number MG264131) was employed as a source of viral inoculum that has been previously characterized [10].

### 2.2. Bacterial Isolation

A nutrient agar (NA) medium with peptone (5%), yeast extract (3%), NaCl (5%), and agar (15%) was used for bacterial isolation [30]. Five tomato rhizosphere samples were collected from healthy tomato fields in Alexandria Governorate, Egypt. Each sample (10 g) was a mix of five (2 g) rhizosphere samples derived from different tomato plants and collected at the same site. After that, each sample (10 g) was shaken in 100 mL of 0.9% NaCl solution for 30 min. Then, 100 µL of each serial dilution was aseptically streaked on duplicate NA plates and incubated at 30 °C for 24 h. Single bacterial colonies from a 10^−6^ dilution were chosen and cultured individually in nutrient broth (agar-free nutrient medium) for 48 h at 30 °C, shaking at 200 rpm. After centrifugation (10 min, 10,000 rpm), the culture filtrate (CF) was collected and filtered with a 0.45 µm pore syringe filter. The purified isolates’ antiviral effects were investigated on *Datura stramonium* plants, serving as TMV local lesion hosts. The upper right half of the leaves received 100 µL of bacterial CF, whereas the left half received 100 µL of sterilized nutrient broth medium. After 24 h, both leaf halves were inoculated with TMV. The study used three biological replicates. The isolate with the highest antiviral activity was chosen for further testing based on its inhibition percentage relative to local lesion numbers.

### 2.3. Molecular Bacterial Identification

The Wizard Genomic DNA Purification Kit (Promega, Fitchburg, WI, USA) was used to purify bacterial genomes following the manufacturer’s instructions. The isolate demonstrating highest antiviral efficacy was molecularly identified using the *16S rRNA*, *rpoB*, and *gyrA* genes (Table 1) and analysis of morphological traits [31]. Sequencing of the purified PCR products was conducted by an Analyzer 3130xl (Applied Biosystems, Foster City, CA, USA), using the BigDye Terminator v3.1 Cycle Sequencing kit (Applied Biosystems, Foster City, CA, USA). Following analyses with NCBI-BLAST, the MEGA 11 software was applied, using UPGMA and a bootstrap method with 2000 replications to uncover the chosen isolate’s phylogenetic relationships. Annotated nucleotide sequences were deposited to GenBank.

### 2.4. Design of Greenhouse Experiments and Evaluation of Growth Parameters

**Figure 1:**
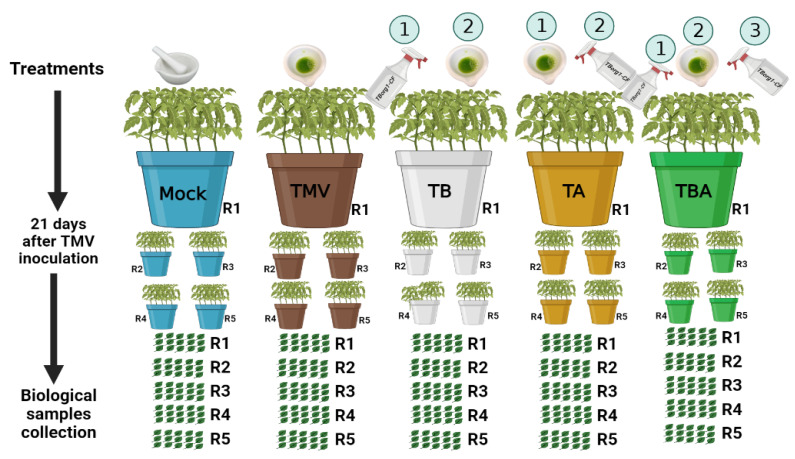
Greenhouse experiment scheme among different treatments. Mock: tomato plants inoculated with viral inoculation buffer; TMV: tomato plants inoculated with TMV; TB: tomato plants sprayed with TBorg1-CF 24 h prior to viral infection; TA: tomato plants sprayed with TBorg1-CF 24 h following viral infection; TBA: tomato plants were sprayed twice, once 24 h before and once 24 h after viral inoculation, with TBorg1-CF.

Seeds of tomato were grown in plastic pots (30 cm in diameter) under greenhouse conditions. Each pot was supplied with 4 kg of autoclaved sterilized sand and clay (1:1). Day and night temperatures of 28 °C/16 °C were used, with a relative humidity of 70% for the incubation of tomato seedlings. One week following transplantation, on the 28th day after sowing, each tomato seedling’s two uppermost true leaves were mechanically inoculated with semi-purified TMV virions (1 mL), as reported previously [32]. Five treatments were used in the experiments, each treatment with five biological replicates and each biological replicate containing five tomato plants per pot (Figure 1). During all analytical evaluations, three uppermost true leaves per tomato plant were used to create a pool of one biological replicate (15 leaves). For every one biological replicate, three technical replicates were performed. The first treatment was the control (mock treatment). The second treatment was TMV inoculation (TMV treatment). The third treatment was tomato foliar treated with bacterial CF 24 h before TMV inoculation (TB treatment). The fourth treatment was tomato foliar treated with bacterial CF 24 h after TMV inoculation (TA treatment). The fifth treatment was tomato foliar treated with bacterial CF 24 h before and after viral infection (TBA treatment). A handheld pressure sprayer was used for spraying the entire plant with bacterial CF until runoff. All plants were kept in insect-proof greenhouses for over three weeks, and the development of mosaic symptoms was investigated daily.

### 2.5. Sample Collection, Disease Assessment, and Determination of Virus Accumulation

Tomato plants from each group were collected at 21 days post-TMV inoculation (dpi), washed many times with running water, weighed, measured, and analyzed for their fresh weight and shoot and root lengths. The plant’s dry weight was calculated after drying at 50 °C. The enzyme activities and gene expression levels were also determined at 21 dpi. Viral accumulation was calculated based on expression levels of the *TMV-CP* gene (Table 1) in plant samples of different treatments. The disease severity was estimated based on visual observation and a rating scale of 0 to 3. While 0 indicates no symptoms, 1 indicates vein clearing; 2 indicates chlorotic and mild mosaic, and 3 indicates severe mosaic and malformation. The disease severity (DS) index was estimated using the following formula:

$$\mathrm{DS}\;\left(\%\right)=\frac{\Sigma \left(\mathrm{disease}\;\mathrm{scale}\times \mathrm{number}\;\mathrm{of}\;\mathrm{plants}\;\mathrm{in}\;\mathrm{each}\;\mathrm{scale}\right)}{\mathrm{total}\;\mathrm{number}\;\mathrm{of}\;\mathrm{plants}\times \mathrm{highest}\;\mathrm{disease}\;\mathrm{scale}}\times 100$$

### 2.6. Oxidative Stress Markers

#### 2.6.1. Determination of Hydrogen Peroxide (H_2_O_2_)

The determination of H_2_O_2_ in the fresh tomato samples was performed using the KI method [33], with a few modifications. A 0.1 g of fresh plant samples were homogenized in 1 mL of 0.1% TCA and centrifuged (10,000 rpm, 15 min, 4 °C) to obtain a clear homogenate. One milliliter of plant homogenate and two milliliters of KI solution (1 M KI in 10 mM potassium phosphate buffer, pH 7.0) were mixed to detect H_2_O_2_. After 20 min, the reaction absorbance was assayed at 390 nm. The results were calculated based on the H_2_O_2_ extinction coefficient (0.28 M^−1^ cm^−1^) and expressed as µmol/g fresh weight.

#### 2.6.2. Determination of Malondialdehyde (MDA)

Thiobarbituric acid (TBA) was used to assay malondialdehyde levels in all treatments, as previously described [34]. One milliliter of 0.1% trichloroacetic acid (TCA) was used to grind 100 mg of tomato leaf samples. The samples were then centrifuged at 10,000 rpm, 30 min at 4 °C, to separate the TCA from the ground-up tomato leaf samples. Each 1 mL of sample supernatant was mixed with 4 mL of TBA solution (0.5% TBA:20% TCA). Then, the samples were put into an oven set at 95 °C for 30 min. The reaction was stopped by immersing the samples in ice. At 600 nm, the color of the product indicated the amount of malondialdehyde present (µmol/g fresh weight).

### 2.7. Measurement of Antioxidant Enzymatic Activities

#### 2.7.1. Polyphenol Oxidase (PPO)

Quinone techniques were used to determine the PPO activity [35]. In summary, 500 mL of crude plant extract was added to 1 mL of 50 mM quinone and incubated at 25 °C for 10 min. The absorbance of the reaction was determined at 420 nm, where a 0.001 rise in absorbance corresponds to one unit of enzyme activity per minute and is reported as µmol/g fresh weight.

#### 2.7.2. Superoxide Dismutase (SOD)

SOD activity was measured using a modified nitroblue tetrazolium (NBT) photoreduction inhibition technique [36]. The crude plant extract (100 µL in 100 mM of potassium phosphate buffer pH 7.0) was combined with the same amount of 50 µM of NBT, 10 µM of riboflavin, 10 mM EDTA, 50 mM sodium carbonate, and 12 µM L-methionine. To obtain a final reaction volume of 3 mL, a 50 mM phosphate buffer, pH 7.6, was added. As controls, reaction mixtures devoid of plant extract were used. After 15 min of exposure to fluorescent lights to commence the photochemical process, the mixtures were put in the dark, and the absorbance at 560 nm was measured. One unit of enzyme activity was defined as a 50% inhibition of the photochemical reduction [37]. SOD activity was quantified as µmol/g fresh weight.

#### 2.7.3. Peroxidase (POX)

POX activity was determined using the technique of Angelini et al. [38]. In summary, 120 µL of 1 mM hydrogen peroxide and 500 µL of 5 mM guaiacol were added to the crude plant extract (80 µL in K-phosphate buffer, pH 7.0). The final volume of 1.2 mL was adjusted by using 100 mM phosphate buffer pH 7.0. The reaction was incubated at 30 °C for 10 min, and the absorbance at 480 nm was determined. Using an extinction coefficient of 26,600 M^−1^ cm^−1^, the POX activity was calculated and expressed as µmol/g fresh weight.

### 2.8. Total Soluble Protein (TSP) Determination

Amounts of total soluble proteins were determined using the Bradford method and a standard curve of bovine serum albumin [39].

### 2.9. Detection of Total Soluble Carbohydrates (TSC)

Total soluble carbohydrate contents (TSC) were determined using the anthrone method, as described previously [40]. Plant leaves were first homogenized in 95% ethanol in a solid: liquid ratio of 1:20. Following precipitation (centrifugation at 5000 rpm for 10 min), 100 mL was mixed with 1 mL anthrone solution (200 mg of anthrone in 100 mL of concentrated H_2_SO_4_) and incubated for 10 min at 100 °C in a water bath. The reaction absorbance was measured at 625 nm after cooling for 1 h. The contents of total soluble carbohydrates (mg/g dry weight) were calculated using a glucose standard curve.

### 2.10. Determination of Ascorbic Acid (AsA)

Ascorbic acid contents were determined using Na-molybdate, as previously reported [41]. Samples of fresh leaves were homogenized in 5% sulfosalicylic acid (solid:liquid ratio of 1:5). The homogenate was then centrifuged at 10,000 rpm and 4 °C for 15 min. A 1 mL of clear leaf extract was added to 5 mL of freshly made reaction mixture consisting of 2% of Na-molybdate, 0.075 M of H_2_SO_4_, and 1.5 mM of Na_2_HPO_4_ (*v*/*v*/*v*). After 40 min of incubation at 60 °C, the absorbance was measured at 660 nm, and the ascorbic acid content (mg/g fresh weight) was determined from an ascorbic acid standard curve.

### 2.11. Free Radical Scavenging Activity Evaluation

The free radical scavenging capacity was investigated according to Shimada et al. [42], as follows: 2 mL 2,2-Diphenyl-1-picrylhydrazyl (DPPH, 0.05 M in methanol) was added to 100 µL of plant leaves extract (in K-phosphate buffer pH 7.0). The color reduction was measured at 517 nm for 30 min and expressed as scavenging activity (%) using the equation: Scavenging of free radicals (%) = (AI − A30/AI) × 100, where AI is the initial reaction absorbance and A30 is the reaction absorbance after 30 min.

### 2.12. Analyzing Results of Quantitative RT-PCR (qRT-PCR)

#### 2.12.1. Extraction and Synthesis of cDNA from Plant Total RNA

One hundred milligrams of tomato leaves were used for total RNA extraction by the guanidium isothiocyanate method [43,44]. The purity and concentration of extracted RNA was measured by a SPECTRO Star Nano instrument (BMG Labtech, Ortenberg, Germany) at A260/A280. Moreover, the quality of 28S and 18S rRNA bands separated by electrophoresis on 1.2% agarose gels was used to assess RNA integrity. In order to synthesize cDNA, 1 μg of DNase I-treated RNA was utilized from each sample as a template in a reverse transcription process. The reaction procedure was carried out with oligo (dT) and random hexamer primers, as was reported in a prior study [45]. The final cDNA product was kept at −20 °C until utilized as a template for qRT-PCR.

#### 2.12.2. Expression of Tomato Defense Genes and TMV-CP

The transcript levels of three tomato genes that encode pathogenesis-related proteins (*PR-1*, *PR-2*, and *PR-5*), and three genes encoding proteins involved in polyphenol metabolism (*C4H*, *HCT*, and *CHI*), along with transcript accumulation of the TMV-coat protein gene (*TMV-CP*) were assessed for all treatments using the qRT-PCR technique. The nucleotide sequences of the primers used are presented in Table 1. Expression values of tomato defense genes and *TMV-CP* were adjusted by assaying the expression of *β-actin* as a reference gene. Expression of *β-actin* was tested during all types of treatments (mock and TMV inoculation with/without CF-treatments), and significant changes in gene expression were not detectable. The qPCR reactions for each biological treatment were performed in a separate batch using a SYBR Green Mix (Thermo Fisher, CA, USA) and run on a Rotor-Gene 6000 real-time thermocycler (QIAGEN, Germantown, MD, USA), as previously described [46]. For each gene studied, relative expression levels were estimated according to the 2^−ΔΔCT^ technique [47].

### 2.13. GC-MS Analysis of Active Biomolecules in Bacterial Culture Filtrates

After ethyl acetate extraction, gas chromatography-mass spectrometry (GC-MS) was employed to detect the active biomolecules in bacterial culture filtrates (CF), as previously reported [15]. Briefly, the CF was added to ethyl acetate in a ratio of 1:1 and shaken vigorously for 20 min, and then phases were separated using a separating funnel. The ethyl acetate phase was concentrated at a temperature of 50 °C using a rotatory evaporator. By using a GC-MS system (TRACE 1300 Series, Thermo, Waltham, MA, USA) with a split mode mass detector and helium as the carrier gas at a flow rate of 1 mL/min, the concentrated CF extracts were screened for secondary metabolite compounds. The injector was set to 250 °C for two minutes and the oven to 60 °C for two minutes with a scan time of 0.2 s; a mass range of 50–650 amu; and a 20-min ramp to 250 °C. During the 53-min run period, mass spectra at 70 eV were recorded. The CF components were identified by comparison to published data and the GC-MS library.

### 2.14. Statistical Analyses

Using the GraphPad Prism software, all data were statistically analyzed using a one-way ANOVA. By using the Tukey’s honest significant differences (H.S.D.) method at a probability value (*p*-Value) ≤ 0.05, significant differences were determined. In the tables and histograms, a column bar is used to show the standard deviation (SD). Compared to mock-inoculated samples, relative gene expression values higher than one depict an increase in transcript accumulation, while values lower than one indicate a decrease in gene expression levels.

## 3. Results

### 3.1. Identification of an Isolate of Bacillus amyloliquefaciens from Tomato Rhizosphere

**Figure 2:**
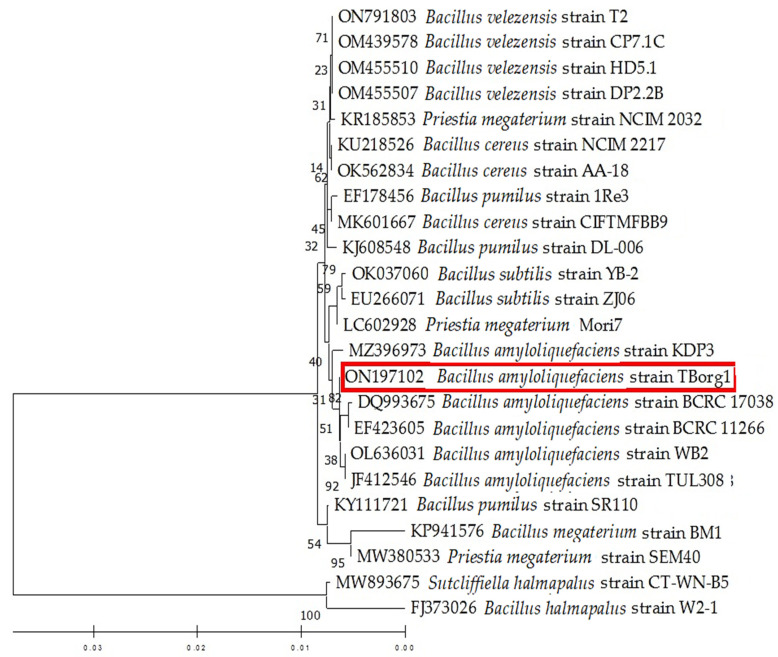
A phylogenic tree depicting the relationships of the isolated *Bacillus amyloliquefaciens* strain TBorg1 (indicated by a red rectangle) and other closely related isolates from GenBank based on the *16s rRNA* nucleotide sequence. The MEGA 11 software used the UPGMA algorithm, and the bootstrap method with 2000 replicates to create the tree.

The morphological investigation of the bacterial isolate that was isolated from the rhizosphere of tomato plants revealed gram-positive and endospore-forming characteristics. The appearance of colonies was creamy-white and rough, and the edges of colonies slightly irregular. PCR reaction results revealed that the amplicons of the three bacterial genes tested, *16S rRNA*, *rpoB*, and *gyrA*, were approximately 1532, 519, and 906 bp in length, respectively. On the basis of NCBI-BLAST alignment and phylogenetic tree analysis, the bacterial isolate was identified as *Bacillus amyloliquefaciens* and deposited in the GenBank database under the name “*Bacillus amyloliquefaciens* strain TBorg1” with the accession numbers of ON197102, ON193513, and ON193514 for the *16S rRNA*, *rpoB*, and *gyrA* genes, respectively. The NCBI-BLAST alignment and phylogenetic tree analysis revealed that the nucleotide sequence of the *16S RNA* gene of TBorg1 (Figure 2) displays a 100% similarity to other *B. amyloliquefaciens* isolates, especially to isolates from India (OL636031 and MZ396973), Taiwan (DQ993675 and EF423605), and Poland (JF412546). On the other hand, the BLAST alignment of the nucleotide sequence of the *gyrA* gene indicated a high similarity (100% identity) to other *B. amyloliquefaciens* isolates, especially to an isolate from Taiwan (CP053376). Furthermore, the nucleotide sequence of the *rpoB* gene exhibited 100% identity with other *B. amyloliquefaciens* isolates deposited in GenBank, particularly with two isolates from China (CP054415 and CP032146).

### 3.2. Effect of TBorg1 Culture Filtrate on Tomato Growth and Systemic Accumulation of TMV

The data of tomato growth parameters from greenhouse experiments showed significant reductions in fresh weight, dry weight, shoot length, and root length of tomato plants that were infected with TMV (TMV treatment), recording 6.89 ± 1.53 g, 1.63 ± 0.16 g, 27.33 ± 1.58 cm, and 9.50 ± 1.51 cm, respectively, as compared to mock-treated plants (Table 2). On the other hand, foliar applications of TBorg1 culture filtrate (CF) either 24 h before, 24 h after, or 24 h before and 24 h after TMV inoculation (TB or TA or TBA) resulted in significantly increased tomato fresh weight, dry weight, shoot length, and root length, in comparison to TMV treatment plants (Table 2). The TBA treatment was the most effective in suppressing the adverse effects of the disease by increasing fresh and dry weights to 10.55 ± 1.38 g and 2.15 ± 0.28 g, respectively. Moreover, shoot length, and root length values following TBA treatments were significantly higher than those of the other treatments, recording 38.50 ± 3.04 cm, and 18.00 ± 3.08 cm, respectively, at 21 dpi.

**Figure 3:**
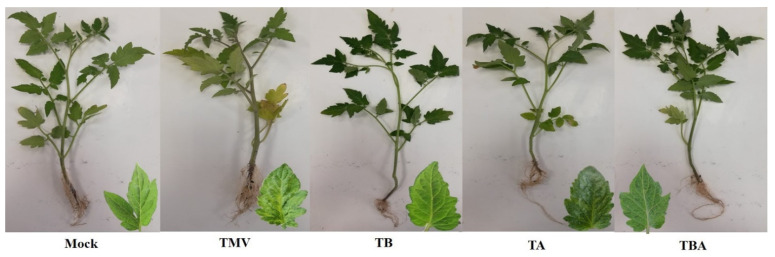
Effect of foliar application of *B. amyloliquefaciens* strain TBorg1-CF on developing disease symptoms in tomato leaves at 18 dpi. Mock: tomato plants inoculated with viral inoculation buffer and foliar sprayed with sterile bacteria-free broth medium; TMV: tomato plants inoculated with TMV and foliar sprayed with sterile bacteria-free broth medium; TB: tomato plants sprayed with TBorg1-CF 24 h prior to viral infection; TA: tomato plants sprayed with TBorg1-CF 24 h following viral infection.; TBA: tomato plants were sprayed twice, once 24 h before and once 24 h after viral inoculation, with TBorg1-CF.

In our greenhouse experiments, we found that the foliar application of *B. amyloliquefaciens* strain TBorg1-culture filtrate (TBorg1-CF) significantly reduces disease severity and decreases virus accumulation in all treated tomato plants (TB, TA, and TBA), as compared to the TMV treatment. The results showed that TMV-inoculated tomato plants developed characteristic symptoms with severe mosaic appearing by 14 dpi (Figure 3). On the other hand, a delay in symptom development of five and three days were found with TB and TA treatments, respectively (Figure 3). Moreover, dual foliar application of TBorg1-CF (TBA) resulted in a ca. 7 days’ delay in symptom development. No disease symptoms were observed on the mock-inoculated plants. Consistent with symptom appearance, the TMV treatment resulted in disease severity of 93.43 ± 1.98% (Table 3). However, TB and TA treatments significantly reduced disease severity to 32.16 ± 1.41% and 43.28 ± 2.43%, respectively (Table 3). Moreover, the TBA treatment exhibited the lowest disease severity of 17.19 ± 1.67%. Importantly, treatments with TBorg1-CF considerably reduced the accumulation of TMV in tomato leaves. Furthermore, qRT-PCR results revealed that the TMV treatment results in the highest levels of *TMV-CP* transcripts, reflecting a relative expression of 27.98, indicating the plant’s viral infection (Table 3). In contrast, the relative expression levels of *TMV-CP* in TBorg1-CF-treated plants were only 4.54, 3.39, and 2.78 for the TA, TB, and TBA treatments, respectively (Table 3). In fact, the small amounts of TMV detected in TBA-treated plants corresponds to a 90% reduction in viral accumulation. These results suggest that TBorg1-CF may indeed confer plant resistance to TMV replication in tomato tissues.

### 3.3. Effect of TBorg1-CF on Oxidative Stress Markers

**Figure 4:**
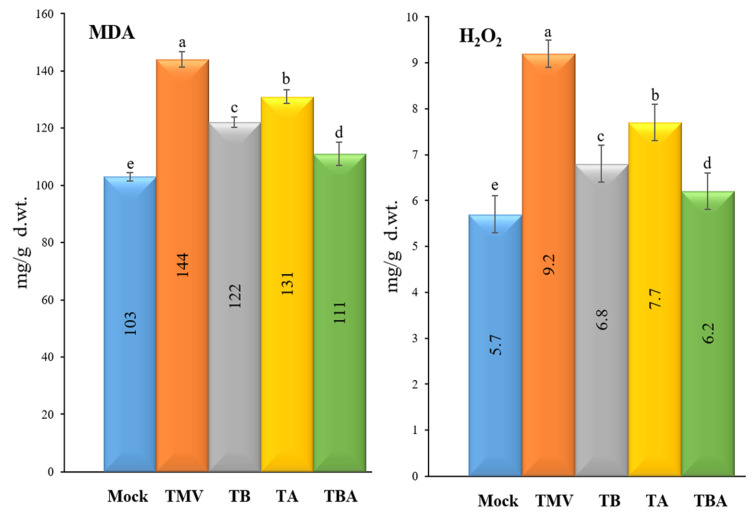
Effect of foliar application of *B. amyloliquefaciens* strain TBorg1-CF on two oxidative stress markers of tomato plants at 21 dpi; MDA (**left**) and H_2_O_2_ (**right**). Mock: tomato plants inoculated with viral inoculation buffer and foliar sprayed with sterile bacteria-free broth medium; TMV: tomato plants inoculated with TMV and foliar sprayed with sterile bacteria-free broth medium; TB: tomato plants sprayed with TBorg1-CF 24 h prior to viral infection; TA: tomato plants sprayed with TBorg1-CF 24 h following viral infection.; TBA: tomato plants were sprayed twice, once 24 h before and once 24 h after viral inoculation, with TBorg1-CF. Each value in a column is the average ± SD of the results from five biological replicates. The average values of the columns with the same letter do not differ significantly.

Levels of two oxidative stress markers, malondialdehyde (MDA) and hydrogen peroxide (H_2_O_2_), were evaluated in the five tomato treatment groups (Figure 4). For MDA, the TMV treatment group showed the highest levels (144 ± 2.8 µmol/g f.wt.), while the mock plants exhibited the lowest MDA levels (103 ± 4.9 µM/g f.wt.). Similarly, the TMV treatment and mock treatment plants showed the highest and lowest levels of H_2_O_2_ with 9.2 ± 0.3 and 5.7 ± 0.4 µmol/g f.wt., respectively (Figure 4). Compared to the TMV treatment, the foliar applications of TBorg1-CF significantly decreased MDA and H_2_O_2_ contents in all treated plants (Figure 4). Among TBorg1-CF treatments, the TBA treatment resulted in the lowest levels of MDA and H_2_O_2_ (111 ± 4.1 and 6.2 ± 0.4 µM/g f.wt.), followed by TB (122 ± 1.8 and 6.8 ± 0.4 µM/g f.wt.), and TA (131 ± 2.4 and 7.7 ± 0.9 µM/g f.wt.), respectively.

### 3.4. Effect of TBorg1-CF on Antioxidant Enzymatic Activities

**Figure 5:**
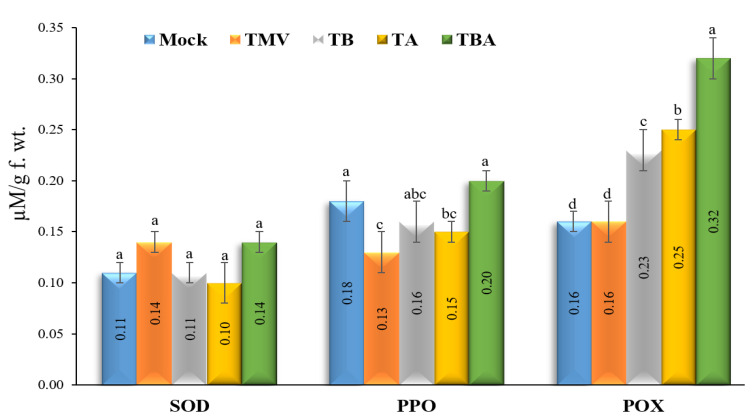
Effect of foliar application of *B. amyloliquefaciens* strain TBorg1-CF on three enzymes exhibiting antioxidant activities—SOD (superoxide dismutase), PPO (polyphenol oxidase), and POX (peroxidase)—in tomato plants at 21 dpi. Mock: tomato plants inoculated with viral inoculation buffer and foliar sprayed with sterile bacteria-free broth medium; TMV: tomato plants inoculated with TMV and foliar sprayed with sterile bacteria-free broth medium; TB: tomato plants sprayed with TBorg1-CF 24 h prior to viral infection; TA: tomato plants sprayed with TBorg1-CF 24 h following viral infection; TBA: tomato plants were sprayed twice, once 24 h before and once 24 h after viral inoculation, with TBorg1-CF. Each value in a column is the average ± SD of the results from five biological replicates. The average values of the columns with the same letter do not differ significantly.

The assay results of the three antioxidant enzymes (SOD, PPO, and POX) revealed that PPO and POX have significantly different activities upon TMV and TBorg1-CF treatments (Figure 5). The results indicated a remarkable increase in POX activates after treatment of tomato plants with TBorg1-CF (Figure 5). Compared to mock treatment (0.16 ± 0.01 µM/g f.wt.), the TBA treatment exhibited the highest POX levels (0.32 ± 0.02 µM/g f.wt.), followed by TA and TB treatments with levels of 0.25 ± 0.01 µM/g f.wt. and 0.23 ± 0.01 µM/g f.wt., respectively. No significant differences in POX activities were found between TMV treatment (0.16 ± 0.02 µM/g f.wt. min^−1^) and mock treatment groups. Regarding PPO (Figure 5), our results showed a significant reduction in PPO activities upon TMV infection in all treatment groups except TBA treatment, which showed a slight increase (0.20 ± 0.03 µM/g f.wt.), with no significant changes as compared to mock treatment (0.18 ± 0.02 µM/g f.wt.). The lowest PPO activities were detected in TMV treatment (0.13 ± 0.01 µM/g f.wt.), followed by TA (0.15 ± 0.01 µM/g f.wt.) and TB (0.16 ± 0.04 µM/g f.wt.) treatments. Regarding SOD, no significant changes in SOD activities were found between different treatments (Figure 5).

### 3.5. Impact of TBorg1-CF on Total Soluble Carbohydrates and Total Soluble Protein Contents

**Figure 6:**
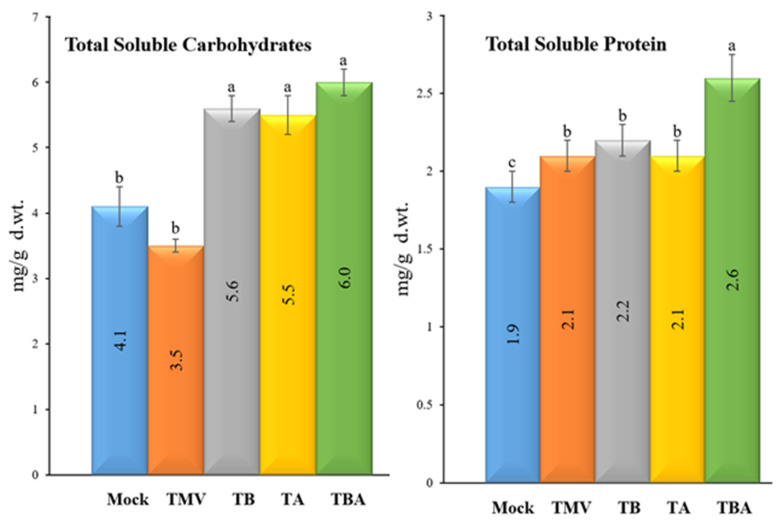
Effect of foliar application of *B. amyloliquefaciens* strain TBorg1-CF on total soluble carbohydrates (**left**) and total soluble protein (**right**) contents of tomato plants at 21 dpi. Mock: tomato plants inoculated with viral inoculation buffer and foliar sprayed with sterile bacteria-free broth medium; TMV: tomato plants inoculated with TMV and foliar sprayed with sterile bacteria-free broth medium; TB: tomato plants sprayed with TBorg1-CF 24 h prior to viral infection; TA: tomato plants sprayed with TBorg1-CF 24 h following viral infection.; TBA: tomato plants were sprayed twice, once 24 h before and once 24 h after viral inoculation, with TBorg1-CF. Each value in a column is the average ± SD of the results from five biological replicates. The average values of the columns with the same letter do not differ significantly.

Regarding the contents of total soluble carbohydrates (TSC), the results revealed that the foliar application of TBorg1-CF significantly elevated TSC contents in the treated tomato plants (Figure 6). The TBA treatment resulted in the highest TSC contents (6.0 ± 0.9 mg/g d.wt.), followed by TA (5.6 ± 0.2 mg/g d.wt.) and TB (5.5 ± 0.3 mg/g d.wt.) treatments, with no significant changes among the three treatments (Figure 6). On the other hand, no significant changes in TSC contents were found between mock (4.1 ± 0.4 mg/g d.wt.) and TMV (3.5 ± 0.1 mg/g d.wt.) treatments. Concerning total soluble protein (TSP) contents, the dual foliar application of TBorg1-CF (TBA treatment) resulted in the highest TSP (2.6 ± 0.2 mg/g d.wt.) among all tomato treatments (Figure 6). Compared to the mock treatment (1.9 ± 0.2 mg/g d.wt.), the TMV, TB, and TA treatment groups exhibited approximately the same TSP contents (2.1 ± 0.1 mg/g d.wt.).

### 3.6. Effect of TBorg1-CF on Free Radical Scavenging Activities and Ascorbic Acid Contents

**Figure 7:**
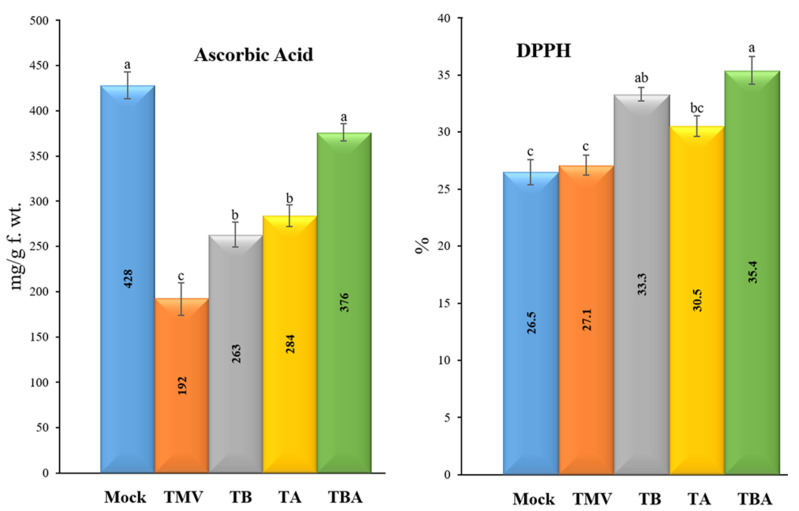
Effect of foliar application of *B. amyloliquefaciens* strain TBorg1-CF on the free radical scavenging activities (**left**) and ascorbic acid contents (**right**) of tomato plants at 21 dpi. Mock: tomato plants inoculated with viral inoculation buffer and foliar sprayed with sterile bacteria-free broth medium; TMV: tomato plants inoculated with TMV and foliar sprayed with sterile bacteria-free broth medium; TB: tomato plants sprayed with TBorg1-CF 24 h prior to viral infection; TA: tomato plants sprayed with TBorg1-CF 24 h following viral infection.; TBA: tomato plants were sprayed twice, once 24 h before and once 24 h after viral inoculation, with TBorg1-CF. Each value in a column is the average ± SD of the results from five biological replicates. The average values of the columns with the same letter do not differ significantly.

The free radical scavenging activities were evaluated using the DPPH method in the five tomato experimental conditions (Figure 7). The results indicated a significant activation of free radical scavenging activities after treatments of tomato plants with TBorg1-CF (TB, TA, and TBA treatments) compared to the mock and TMV treatments (Figure 7). The TBA treatment group showed the highest DPPH levels/free radical scavenging activities (35.4 ± 3.2%), followed by TB (33.3 ± 1.2%) and TA (30.5 ± 0.9%) treatments. No significant differences were found between the TMV (27.1 ± 1.3%) and mock (26.5 ± 2.3%) treatments (Figure 7). Remarkably, ascorbic acid contents were significantly reduced upon TMV infection (to 192 ± 9.5 mg/g f.wt.) as compared to the mock treatment (428 ± 18.1 mg/g f.wt.) (Figure 7). In comparison to the TMV treatment, the treatment of tomato plants with TBorg1-CF either before (TB treatment) or after (TA treatment) TMV inoculation considerably increased ascorbic acid contents to 263 ± 16.4 mg/g f.wt. and 284 ± 19.7 mg/g f.wt., respectively, (Figure 7). Interestingly, no significant changes were found in ascorbic acid contents between the TBA treatment (376 ± 21.3 mg/g f.wt.) and the mock treatment (428 ± 18.1 mg/g f.wt.) (Figure 7).

### 3.7. Effect of TBorg1-CF on the Expression of Tomato Defense Genes

#### 3.7.1. Polyphenol Biosynthetic Pathway Genes

**Figure 8:**
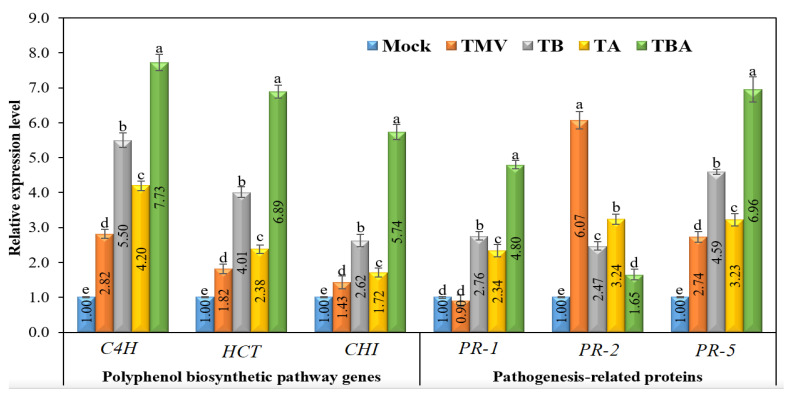
Effect of foliar application of *B. amyloliquefaciens* strain TBorg1-CF on the expression of genes encoding polyphenol biosynthetic pathway enzymes (cinnamate 4-hydroxylase, C4H; hydroxycinnamoyl transferase, HCT; chalcone isomerase, CHI) and pathogenesis-related (PR-1, PR-2 and PR-5) proteins of tomato plants at 21 dpi. Mock: tomato plants inoculated with viral inoculation buffer and foliar sprayed with sterile bacteria-free broth medium; TMV: tomato plants inoculated with TMV and foliar sprayed with sterile bacteria-free broth medium; TB: tomato plants sprayed with TBorg1-CF 24 h prior to viral infection; TA: tomato plants sprayed with TBorg1-CF 24 h following viral infection.; TBA: tomato plants were sprayed twice, once 24 h before and once 24 h after viral inoculation, with TBorg1-CF. Each value in a column is the average ± SD of the results from five biological replicates. The average values of the columns with the same letter do not differ significantly.

The transcript levels of three genes (*C4H*, *HCT*, and *CHI*) encoding the critical enzymes that regulate polyphenol biosynthesis pathways were studied at 21 dpi after TMV inoculation. (Figure 8). Compared to the mock treatment (control), a significant up-regulation of *C4H* in the four tomato treatment groups (TMV, TB, TA, and TBA) was observed (Figure 8). The qPCR results revealed that the viral infection, as well as TBorg1-CF applications, induced transcript levels of the *C4H* gene. The TBA treatment group exhibited the highest expression levels, with a relative expression of 7.73, compared to the control group. The TMV treatment showed a relative expression of 2.82, while TB and TA treatments exhibited relative expressions of 5.50 and 4.20, respectively (Figure 8). Similarly to *C4H*, *HCT* was also induced in all tomato treatments compared to the control (Figure 8). A significant up-regulation of *HCT* was found with relative expression levels of 1.82, 2.38, and 4.01 for TMV, TA, and TB treatments, respectively. The TBA treatment exhibited the highest *HCT* transcript levels with a relative expression 6.89-fold higher than the control.

Regarding *CHI*, despite the up-regulation of the *CHI* gene in all treatments compared to the control, it exhibited the lowest transcript levels compared to *C4H* and *HCT* (Figure 8). Nevertheless, the dual foliar application of TBorg1-CF resulted in the highest expression levels of *CHI* with a relative expression of 5.74, followed by TB and TA treatments with relative transcript levels of 2.62 and 1.72, higher than that of the control. Even the TMV treatment group displayed a significant change in *CHI* expression level of 1.43-fold compared to the mock (control) treatment (Figure 8).

#### 3.7.2. Pathogenesis-Related Protein-Encoding Genes

Compared to the control (mock), a clear difference in transcriptional profiles of three genes (*PR-1*, *PR-2*, and *PR-5*) encoding three pathogenesis-related proteins was observed (Figure 8). Concerning *PR-1*, its expression was induced only in TBorg1-CF treated plants with relative expressions of 2.76 and 2.34 in TB and TA treatments, respectively (Figure 8). The dual TBorg1-CF application treatment (TBA) showed the best results of *PR-1* gene induction with a 4.80-fold higher expression than the mock treatment. The TMV treatment group exhibited a slight decrease in *PR-1* relative expression (0.90-fold) compared to the mock treatment (Figure 8). For *PR-5* expression, a significant up-regulation (2.74-fold) was observed in TMV-treated plants compared to the mock plants. However, in plants treated with TBorg1-CF either before (TB) or after (TA) infection, expression levels of *PR-5* were 4.59- and 3.23-fold higher, respectively, than that of the control (Figure 8). The TBA treatment group showed the highest induction of *PR-5* expression, with transcript levels 6.96-fold higher than in the mock treatment group. Thus, treatments with either TMV or TBorg1-CF can effectively trigger *PR-5* expression. Regarding *PR-2*, the TMV treatment group showed the most significant up-regulation of expression with a 6.07-fold higher value than in the mock treatment (Figure 8). However, compared to the TMV treatment, the TBorg1-CF treatment groups (either before or after TMV inoculation or dual CF application) exhibited a significant reduction in transcript levels of *PR-2* (Figure 8). The TB and TA treatments resulted in relative expression levels of 2.47 and 3.24, respectively, while the TBA treatment group showed a relative expression level only 1.65-fold higher than that of the control (Figure 8).

### 3.8. GC-MS Analysis of Bioactive Metabolites of TBorg1-CF

Using a GC-MS equipment, the bioactive components of the ethyl acetate extract of TBorg1-CF were identified. The active ingredients, including their retention time (RT), peak height, chemical formula, molecular weight, and molecular structures are shown in Table 4. Among the 15 bioactive compounds detected in the GC-MS analysis, 1,2-benzenedicarboxylic acid mono(2-ethylhexyl) ester showed the highest peak area with an RT of 23.369, followed by the compounds phenol, 2,4-bis(1,1-dimethylethyl)- and L-proline, N-valeryl-, heptadecyl ester at RTs of 12.189 and 15.542, respectively (Table 4). The compounds n-hexadecanoic acid, pyrrolo[1,2-a]pyrazine-1,4-dione hexahydro-3-(2-methylpropyl)-, as well as Nonane, 5-butyl-, and eicosane displayed moderate peak areas at RTs of 15.483, 15.437, 13.690, and 15.232, respectively. Moreover, hexadecane, tetradecane, and 1-tridecene were also detected at RTs of 12.795, 12.745, and 14.317, respectively. Other constituents displayed varying retention times and peak areas.

## 4. Discussion

Plant viruses are among the most significant plant disease-causing agents, since over half of all newly developing epidemics have a viral etiology [3,16]. This creates issues regarding food security, and is also accountable for enormous losses in crop productivity. Chemical treatments, such as pesticides and insecticides, must be monitored and controlled due to adverse effects on human health and environmental risks. In agriculture, biological pest management utilizing PGPR (one or more strains) is being evaluated as an improvement over chemical control for fighting plant diseases [48]. Therefore, there is an urgent need for research on novel, environmentally acceptable biocontrol agents that can manage viral plant diseases. The current study evaluated the antiviral activities of a newly identified *B. amyloliquefaciens* strain, TBorg1, against TMV in tomato plants. The morphological features of the TBorg1 strain were consistent with the description of *B. amyloliquefaciens* in Bergey’s Manual of Systematic Bacteriology [49]. Furthermore, sequence analyses of representative bacterial genes (*16S rRNA*, *rpoB*, and *gyrA*) of TBorg1 confirmed morphological investigations. However, instead of using the PGPR soil application method for our antiviral biocontrol strategy, we sprayed tomato shoots with the CF of the strain TBorg1, since microbial CFs are regarded as an environmentally friendly and dependable alternative to PGPRs [20]. We have demonstrated that tomato plants treated with TBorg1-CF display a reduced rate of TMV infection, with significantly milder systemic symptoms and disease severity that correlates with the suppression of virus accumulation in tomato leaf tissues. These results demonstrate the efficacy of TBorg1-CF in controlling the disease caused by TMV in tomatoes.

Under greenhouse conditions, the foliar application of TBorg1-CF in TMV-infected tomatoes significantly increased the shoot and root lengths and the fresh weights (TB, TA, and TBA treatments) compared to TMV-infected plants without CF treatments. These findings demonstrate that TBorg1 can significantly improve the growth and development of plant roots and shoots, exhibiting the characteristics of PGPR. Interestingly, the dual application of TBorg1-CF (TBA treatment) resulted in a 7-day delay in TMV-elicited symptoms compared to untreated tomato, which developed severe mosaic symptoms by 14 dpi, as previously described [7,12]. Several authors reported that the foliar application of bacterial culture filtrates results in delayed development of plant viral symptoms [7,15,16,50]. Consistent with symptom appearance, the TA, TB, and TBA treatments significantly reduced TMV disease severity to 43.28, 32.16, and 17.19%, respectively. Furthermore, treatments with TBorg1-CF considerably reduced TMV accumulation by up to 90% in the TBA treatment. Thus, the foliar application of TBorg1 may be capable of mediating induced resistance against TMV in tomato plants. Foliar applications of biological control agents are less widespread than soil treatments, and numerous studies have revealed the effectiveness of *B. amyloliquefaciens* in increasing the growth of numerous plant species, mediated through a large variety of growth-promoting secondary metabolites and phytohormones [51,52,53]. These findings imply that the CF of *B. amyloliquefaciens* TBorg1 may contain secondary metabolites that directly suppress TMV and/or play a significant role in inducing SAR. In this context, the foliar application of *B. amyloliquefaciens* Ba33 resulted in the suppression of TMV and tomato yellow leaf curl virus in tobacco and tomato plants [54,55].

It has been demonstrated that the SAR enhances the production of antioxidant protective enzymes, secondary metabolites, and the expression of plant defense-related genes [16,25]. In the present study, the potential of TBorg1-CF was assessed to induce the activities of antioxidant enzymes and reduce oxidative stress in tomato plants during TMV infection. The considerable increase in H_2_O_2_ and MDA in TMV-infected tomato was compatible with the characteristics of viral-infected plants and a high level of reactive oxygen species [56,57]. Interestingly, the foliar applications of TBorg1-CF could be associated with a significant decrease in both oxidative stress markers. The suppression of activities of oxidative stress-generating enzymes was found to maintain the integrity and stability of cell membranes [58]. Moreover, the foliar application of the culture filtrate of *B. amyloliquefaciens* QSB-6 improved plant stress tolerance by inhibiting MDA buildup in plant tissues [59]. Thus, the considerable decrease in MDA and H_2_O_2_ levels demonstrates the efficiency of TBorg1-CF in mitigating oxidative stress in virus-infected plants. Similarly, the increased activities of enzymes with antioxidant functions such as POX and PPO in leaves of virus-infected tomato plants limited tissue oxidation and pathogen penetration by strengthening cell walls [60]. In our study, TMV infection induced a considerable reduction in PPO activity compared to the mock treatment. In contrast, PPO and POX enzyme activities achieved their maximum levels in TBA treatment plants. These alterations indicate that these enzymes might play an important role in ROS detoxification in tomato plants after TBorg1-CF treatment [61,62]. Furthermore, our results imply that the enhancement of PPO and POX enzyme activities by TBorg1-CF could contribute to the arrest of TMV in tomato cells by promoting the establishment of polymerized phenolic barriers around infection sites [63,64]. Activities of such enzymes (limiting tissue oxidation and pathogen penetration) were significantly increased in virus-infected plants after being exposed to culture filtrates of biocontrol agents that cause SAR [57,59]. Moreover, the application of TBorg1-CF significantly increased total soluble carbohydrates, total soluble protein contents, and free-radical quenching activity. Ascorbic acid is a primary non-enzymatic antioxidant essential for plant development and defense. However, the exact mechanism of how ascorbic acid may enhance plants’ resistance to infection is still unclear [65,66]. Compared to TMV infection alone, the TB, TA, and TBA treatments significantly increased the ascorbic acid contents of tomato tissues. An increased accumulation of ascorbic acid in plant tissues alleviates symptoms of viral infection and inhibits RNA virus replication [67,68]. Regarding free radical scavenging activity (DPPH), we have observed that TB and TBA treatments significantly enhance DPPH activity in treated and TMV-infected tomato tissues compared to TMV and mock treatments. It has been reported that increasing the plant’s free radical scavenging potential is part of its defense to microbial infections to mitigate the negative side effects of the surge in oxidative stress [69,70]. The obtained data demonstrate that *B. amyloliquefaciens* TBorg1 and other beneficial bacteria share comparable strategies for controlling plant viral infections [51,55,57].

Gene expression analysis revealed that the relative expression levels of *C4H*, *HCT*, and *CHI* were significantly increased in all treatments compared to the mock treatment. As secondary metabolites, plants’ polyphenolic compounds are important for plant growth and defence against biotic and abiotic stressors. [16,71]. *C4H* encodes one of the most important enzymes in the phenylpropanoid biosynthetic pathway that plays a vital role in converting cinnamic acid to *p*-coumaric acid, followed by the formation of the main intermediate for polyphenolic compounds [72]. *HCT* is the first enzyme in the chlorogenic acid pathway that catalyzes the conversion of *p*-coumaroyl CoA to shikimate, which results in chlorogenic acid production [73]. *CHI* is one of the most important enzymes in the flavonoid pathway. It converts naringenin chalcone into naringenin, a key step in synthesizing additional flavonoid compounds [72]. The up-regulation of such genes in infected tomato tissues reflects their role in defense against viral infection. The highest transcriptional levels were shown in the TBA treatment with relative expression levels of 5.74-, 6.89-, and 7.73-fold, followed by TB treatment, with 2.62-, 4.01-, and 5.50-fold higher than the control for *CHI*, *HCT*, and *C4H*, respectively. The activation of such defense-related tomato genes in TB, TA, and TBA treatments suggests that TBorg1-CF is an effective elicitor of SAR that is linked to biosynthesis and accumulation of polyphenolic compounds in tomato tissues. Moreover, the very high transcript levels of these genes detected by us in the TBA treatment may correspond to the high levels of flavonoid accumulation in plant tissues, suggesting the significance of treatment dosages (dual foliar application) relative to treatment time (single foliar application) in the accumulation of these defense-related compounds [57].

Many reports state that the activation of several PR proteins plays an essential role in activating SAR, and is also effective in reducing pathogen development, multiplication, or dissemination of pathogens [74,75,76]. In line with these observations, the foliar applications of TBorg1-CF, either as TB, TA, or TBA treatments, resulted in a considerable increase in the transcript levels of the *PR-1*, *PR-2*, and *PR-5* genes. Compared to the mock treatment, the transcript accumulation of *PR-1* was significantly induced, with relative expression levels of 2.34, 2.76, and 4.80 in TA, TB, and TBA treatments, respectively. The elevation of *PR-1* expression is directly correlated with the activation of salicylic acid during pathogen infection [25,77]. The *PR-5* gene encodes thaumatin-like proteins and is involved in the host defense system against biotic and abiotic challenges, and also in regulating physiological processes in several plant species [78]. *PR-5* transcriptional profiles in the current study were similar to *PR-1*, where the expression levels were induced upon TMV challenge during different treatments. TMV infection alone caused a relative expression of *PR-5* 2.74-fold greater than that of the control. These results agree with a previous report showing that infection of *Arabidopsis thaliana* with beet severe curly top virus boosted *PR-5* expression levels [79]. Moreover, tobacco plants infected with tobacco vein banding mosaic virus also exhibited an up-regulation of the *PR-5* gene [80]. In the present study, the foliar application of TBorg1-CF enhanced the expression of *PR-5*, resulting in maximum expression levels during the TBA treatment (6.96-fold change compared to the control). Therefore, these results suggest that TBorg1-CF could be used as a plant systemic immunity mediator by greatly boosting the expression of SA-inducible genes, which are all considered vital SAR markers [27,76,81]. Regarding the expression of *PR-2*, our results demonstrated a significant induction of *PR-2* in TMV-infected plants, with a relative expression level 6.07-fold greater than in the control. Although *PR-2* was up-regulated in TBorg1-CF-treated plants, a significantly lower expression was observed than in TMV infection without TBorg1-CF-treatments. The *PR-2* gene encodes β-1,3-glucanase, which may allow viruses to move between plant cells via plasmodesmata. Therefore, TMV might activate this gene to facilitate its mobility and spread within plant cells [10,82], consistent with previous research that found a marked induction of *PR-2* during viral infections in potato, *Arabidopsis*, onion, tobacco, and tomato plants [7]. In addition, the absence of tobacco *PR-2* expression decreased sensitivity to viral infection, while the overexpression of *PR-2* accelerated the cell-to-cell transmission of potato virus Y [83,84,85]. Foliar treatment of TBorg1-CF may reduce TMV infection by, e.g., inhibiting the cell-to-cell and long-distance movement of the virus by lowering *PR-2* expression.

Microbial secondary metabolites are precursors to many biological functions; therefore, monitoring these compounds by various analytical techniques is critical for understanding biological regulation for novel applications [86]. With the aid of a GC-MS instrument, bioactive components in an ethyl acetate extract of TBorg1-CF were identified in the current study. According to the GC-MS analysis results, TBorg1-CF contains 15 distinct components. 1,2-Benzenedicarboxylic acid, mono(2-ethylhexyl) ester was present in the highest concentration, followed by the compounds phenol, 2,4-bis(1,1-dimethylethyl)- and L-proline, N-valeryl-, heptadecyl ester. In addition, n-hexadecanoic acid, pyrrolo[1,2-a]pyrazine-1,4-dione hexahydro-3-(2-methylpropyl)-, nonane, 5-butyl-, and eicosane were detected at moderate levels. The results were similar to those previously reported for the culture filtrates of several *Bacillus* spp. [15,16,57]. Under fungal and bacterial attack, phenol 2,4-bis(1,1-dimethylethyl)- accumulates in plant cells. It is a compound playing a significant role in plant disease resistance, possibly by inhibiting reactive oxygen species (ROS) [87,88]. TMV infection is frequently associated with elevated ROS levels; thus, inhibiting ROS may alleviate the symptoms of viral infection, as shown previously for infections by, e.g., TMV and other plant viruses [70,89]. Eicosane (a long-chain fatty acid) is a biologically active compound derived from different microorganisms and is a potent antimicrobial for controlling different plant pathogens [90,91]. Pyrrolo[1,2-a]pyrazine-1,4-dione, hexahydro-3-(2-methylpropyl)- is a heterocyclic compound with various biological activities, including antimicrobial, antiviral, anti-inflammatory, and anticancer properties [92,93,94,95]. According to a recent study, the extract of *B. velezensis* contains several different pyrrolo[1,2-a]pyrazine-1,4-dione compounds, which inhibit *Fusarium oxysporum* and increase the systemic resistance to cucumber mosaic virus [15]. It has been found that the mono(2-ethylhexyl) ester of benzenedicarboxylic acid is produced by marine *Streptomyces* spp. and exerts anticancer action in vitro against human breast adenocarcinoma and hepatocellular liver carcinoma [96]. In summary, the findings of our GC-MS analysis indicated the presence of several physiologically active compounds in the supernatant of *B. amyloliquefaciens* strain TBorg1 with different antimicrobial properties. Thus, TBorg1 can be suggested as a potentially powerful PGPR for use in agricultural pest management. However, additional research is needed to determine the exact mechanism(s) underpinning the antiviral activity of TBorg1 towards plant viruses such as TMV.

## 5. Conclusions

The rhizobacterium *B. amyloliquefaciens* strain TBorg1-CF was found to be an effective growth promoter and tomato defense stimulator against TMV infection. Under greenhouse conditions, the foliar applications of TBorg1-CF significantly improved the growth parameters of tomato (shoot and root length, fresh weight), decreased TMV disease severity, and reduced virus levels by up to 90%. Furthermore, considerable increases in total soluble proteins, total soluble carbohydrates, ascorbic acid, and activities of enzymes capable of scavenging reactive oxygen species (PPO and POX), as well as significantly decreased amounts of non-enzymatic oxidative stress markers (H_2_O_2_ and MDA), compared to untreated plants, were also observed. In addition, enhanced systemic resistance to TMV was associated with increased transcript levels of genes encoding polyphenolic pathway enzymes and pathogenesis-related (PR) proteins. The main antimicrobial compounds detected in the TBorg1-CF ethyle acetate extract were 1,2-benzenedicarboxylic acid mono(2-ethylhexyl) ester, phenol-2,4-bis(1,1-dimethylethyl)-, L-proline-N-valeryl-heptadecyl ester, and pyrrolo[1,2-a]pyrazine-1,4-dione hexahydro-3-(2-methylpropyl)-. Our findings indicate that the newly isolated *B. amyloliquefaciens* strain TBorg1 is a promising source of plant growth promotion and antiviral compounds for effectively managing plant diseases.

## Figures and Tables

**Table 1:** The nucleotide sequences of the specific primers that were used in this study.

Primer and Gene Name	Abbreviation	Direction	Nucleotide Sequence
16S ribosomal RNA	*16S rRNA*	Forward	AGAGTGATCCTGGCTCAG
		Reverse	GGTTACCTTGTTACGACTT
RNA polymerase subunit beta	*rpoB*	Forward	CGTGTTATCGTTTCCCAGC
		Reverse	AAGATGATCGATATCATCTG
DNA gyrase subunit A	*gyrA*	Forward	CAGTCAGGAAATGCGTACGTCCTT
		Reverse	CAAGGTAATGCTCCAGGCATTGCT
*Tobacco mosaic virus*-coat protein	*TMV-CP*	Forward	ACGACTGCCGAAACGTTAGA
		Reverse	CAAGTTGCAGGACCAGAGGT
Pathogenesis related protein-1	*PR-1*	Forward	GTTCCTCCTTGCCACCTTC
		Reverse	TATGCACCCCCAGCATAGTT
β-1,3-glucanase	*PR-2*	Forward	TATAGCCGTTGGAAACGAAG
		Reverse	CAACTTGCCATCACATTCTG
Thaumatin-like protein	*PR-5*	Forward	ATGGGGTAAACCACCAAACA
		Reverse	GTTAGTTGGGCCGAAAGACA
Cinnamate 4-hydroxylase	*C4H*	Forward	CCCAGTTTTTGGAAATTGGCTTCA
		Reverse	GCCCCATTCTAAGCAAGAGAACATC
Hydroxycinnamoyl transferase	*HCT*	Forward	TCTCCAACCCCTTTTAACGAACC
		Reverse	CAACTTGTCCTTCTACCACAGGGAA
Chalcone isomerase	*CHI*	Forward	CACCGTGGAGGAGTATCGTAAGGC
		Reverse	TGATCAACACAGTTGGAAGGCG
*β*-actin	*β-actin*	Forward	TGGCATACAAAGACAGGACAGCCT
		Reverse	ACTCAATCCCAAGGCCAACAGAGA

**Table 2:** Effects of foliar application of *B. amyloliquefaciens* strain TBorg1 culture filtrate on tomato plant growth parameters at 21 dpi.

Treatment *	Fresh Weight (g/Plant)	Dry Weight (g/Plant)	Shoot Length (cm)	Root Length (cm)
Mock	8.46 ± 1.82 b	1.91 ± 0.09	33.00 ± 3.04 b	14.67 ± 2.05 b
TMV	6.89 ± 1.53 e	1.63 ± 0.16	27.33 ± 1.58 d	9.50 ± 1.51 e
TB	9.32 ± 1.74 c	1.99 ± 0.21	31.00 ± 2.65 c	13.00 ± 1.65 c
TA	7.32 ± 1.76 d	1.81 ± 0.22	27.67 ± 3.54 d	12.00 ± 2.12 d
TBA	10.55 ± 1.38 a	2.15 ± 0.28	38.50 ± 3.04 a	18.00 ± 3.08 a

* Mock: tomato plants inoculated with viral inoculation buffer and sprayed with sterile bacteria-free broth medium; TMV: tomato plants inoculated with TMV and foliar sprayed with sterile bacteria-free broth medium; TB: tomato plants sprayed with TBorg1-CF 24 h prior to viral infection; TA: tomato plants sprayed with TBorg1-CF 24 h following viral infection.; TBA: tomato plants were sprayed twice, once 24 h before and once 24 h after viral inoculation, with TBorg1-CF. Each value in a column is the average ± SD of the results from five biological replicates. The average values of the columns with the same letter do not differ significantly.

**Table 3:** Effects of foliar application of *B. amyloliquefaciens* strain TBorg1-CF on disease severity and accumulation levels of *TMV-CP* gene in tomato plants at 21 dpi.

Treatments	Disease Severity		Relative Expression Level of *TMV-CP* Gene	
	Value (%)	Decrease (%)	Level	Decrease (%)
Mock	00.00 ± 0.00 e	100	01.00 ± 0.00 e	96.43
TMV	93.43 ± 1.98 a	-	27.98 ± 2.54 a	-
TB	32.16 ± 1.41 c	65.58	03.39 ± 0.21 c	87.88
TA	43.28 ± 2.43 b	53.68	04.54 ± 0.16 b	83.77
TBA	17.19 ± 1.67 d	81.60	02.78 ± 0.13 d	90.06

The average values of the columns with the same letter do not differ significantly.

**Table 4:** The chemical characteristics of the bioactive components in the ethyl acetate extract of *B. amyloliquefaciens* strain TBorg1-culture filtrate as analyzed by gas chromatography–mass spectrometry (GC–MS).

Retention Time (min)	Peak Area	Peak Height	Name	Chemical Formula	Molecular Weight	Molecular Structure
11.961	463.45	31,053	Nonane, 5-(2- methylpropyl)-	C_13_H_28_	184	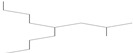
12.189	1384.35	81,171	Phenol, 2,4-bis(1,1-dimethylethyl)-	C_14_H_22_O	214	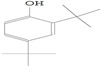
12.334	142.32	10,468	Undecane, 3,7-dimethyl-	C_13_H_28_	184	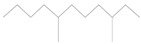
12.745	164.58	10,340	1-Tridecene	C_13_H_26_	182	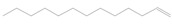
12.795	258.18	16,484	Hexadecane	C_16_H_34_	266	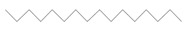
13.690	492.23	31,696	Nonane, 5-butyl-	C_13_H_28_	184	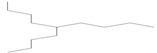
14.012	107.14	8246	Decane, 3,7-dimethyl-	C_12_H_26_	170	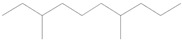
14.275	119.90	9050	1-Hexadecanol	C_16_H_34_O	242	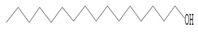
14.317	219.01	15928	Tetradecane	C_14_H_30_	198	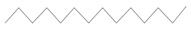
15.232	416.81	24,979	Eicosane	C_20_H_42_	282	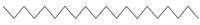
15.400	309.24	17,950	7,9-Di-tert-butyl-1-oxaspiro(4,5)deca-6,9-diene-2,8-dione	C_17_H_24_O_3_	276	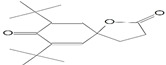
15.437	774.62	44,014	Pyrrolo[1,2-a]pyrazine-1,4-dione, hexahydro-3-(2-methylpropyl)-	C_11_H_18_N_2_O_2_	210	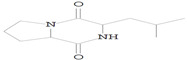
15.483	942.51	39,137	n-Hexadecanoic acid	C_16_H_32_O_2_	256	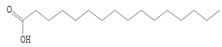
15.542	1.150.21	69,842	L-Proline, N-valeryl-, heptadecyl ester	C_27_H_51_NO_3_	437	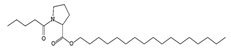
23.369	143.985.58	2,461,918	1,2-Benzenedicarboxylic acid, mono(2-ethylhexyl) ester	C_16_H_22_O_4_	278	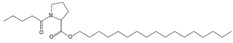

## Data Availability

Not applicable.
